# Supplementary material for: Identification of COVID-19 spread mechanisms based on first-wave data, simulation models, and evolutionary algorithms
Source: PLoS One. 2022 Dec 28;17(12):e0279427. doi: 10.1371/journal.pone.0279427 (PMC9797101; doi:10.1371/journal.pone.0279427)
Supplement: Attachment — Submitted filename: Answers_plos_one_revision.docx [file pone.0279427.s001.docx]

We would like to thank the reviewers for their insightful suggestions. We have tried our best to address them.

We made the source code, as well as initial data, parameters, errors and generated graphs publicly available in a Github repository: <https://github.com/VladimirStanovov/OWID-COVID-19-Analysis>

Reviewer #1

**1. It is well known that a system of simultaneous nonlinear differential equations is very sensitive in terms of its initial conditions. However, the initial conditions information is missing.**

The initial conditions of the models, i.e. state variables, were set according to the available data. First value of the Infected, that were recorded in official statistics, were used for initialization of the Infected state. Number of susceptible was determined according to the population size of each country. In particular, in addition to the mentioned parameters, for every model the algorithm had to tune the $\omega$ parameter, responsible for the total number of susceptible at the considered time period. For example, if the total population was 1 million people, setting $\omega=0.01$ meant that the number of susceptible was set to 10000. Other states, such as exposed, recovered and unreported, were initialized to zero.

**2. It used a heuristic algorithm to estimate the beginning and the end of the first wave. This algorithm uses some arbitrarily taken parameters. It was not clear if the results are robust by changing these parameters.**

Several experiments were conducted to develop and parameterize the proper mechanism to identify first wave. The algorithm was manually checked for all countries. We have added Figure 14 with a short analysis of the influence of $\tau$ and *wp* parameters, controlling the algorithm for determining the first wave.

One should consider that data for some countries were obviously not taken in regular intervals and some irregularities in number of infected cases also occur, like decrease in cumulative number of infected which should not be possible. This was the reason that a general heuristic approach was designed and used for all countries.

The two main parameters controlling the proposed heuristics for determining the first wave are $\tau$ and $wp$. To demonstrate the influence of these values, Figure 14 shows the possible scenarios of determining the first wave with varied parameter values.

**3. All extended versions of the well-known SI, SIR, SEIR, and SEUIR models add the new parameter theta. The original SI, SIR, SEIR, and SEUIR models are particular cases of these extended versions when theta goes to zero. So, by construction, the predictive power of the extended versions needs to be at least as good as the original versions (assuming that the same initial condition was used). However, in Table 4, there are some cases when the extended version is worse. This contradiction probably arises from two origins: (i) some limitations of the optimization algorithm itself; (ii) the original and extended versions use different initial conditions. This must be better explained, and a better fitting process must be made.**

The optimization algorithm used in the experiments starts with a random set of solutions, and although the search ranges, starting and ending dates are the same, the final results are always different. The search process for standard and modified models is performed in search spaces with different dimensions. Adding more variables results in more local optima.

We have also observed this before the paper submission, however, we considered, that the main information lies in the model structure which has been identified and it’s connected to dynamics and offers certain explanation about the process.

We went further to the fine-tuning of the optimization process. An additional series of experiments has been performed to test if it is possible to improve the results by fine-tuning the standard models. This fine-tuning means that the parameters of the standard model, such as SI, SIR, SEIR or SEIUR are taken as used as a starting point in the population of the DE individuals to search for optimal parameter values of the modified models, the resulting models are marked as BD_FT_, eSIR_FT_, eSEIR_FT_ and eSEIUR_FT_. Note that only one of the individuals in DE population was initialized using parameters of standard models, and all other individuals were initialized randomly. The results of these additional experiments have shown that fine-tuning gives either better or at least the same error rates.

**4. In the fitting process, the parameters are optimized within some boundaries taken as given. It was not explained why these boundaries are chosen and how robust are the results by changing their values.**

The ranges for $\sigma$ and $\gamma$ were chosen according to [26], where transmission from exposed to infected and from infected to recovered were determined between 3 and 14 days. For $\beta$ the search range was set from 0 to 4 according to preliminary experiments and literature, for example in [27] the $\beta$ parameter was set to from 1.5 to 3.

To estimate whether the ranges of the parameters' search are appropriate, the boxplots were drawn for the $\beta$ parameter for all considered models. The $\beta$ parameter was chosen, as it is present in all models, and has significant influence, as it is responsible for new infections caused by contacts between infected and susceptible. Figure 16 shows box plots for all models, the parameter values were taken from all 10 runs on all countries.

26 Chu, J. A statistical analysis of the novel coronavirus (COVID-19) in Italy and Spain. *PLoS ONE,* 2021, *16*, <https://doi.org/10.1371/journal.pone.0249037>
27 Schmitt, F.G. An algorithm for the direct estimation of the parameters of the SIR epidemic model from the I(t) dynamics. *European Physical Journal plus,* 2022*,* 137, <https://doi.org/10.1140/epjp/s13360-021-02237-7>

**5. The goodness-of-fit is measured using a normalized version of the Euclidean distance between the actual and the predicted cumulative number of cases. It would be interesting to verify the robustness of the results with other metrics.**

Preliminary, we carefully examined the fitting process by using several standard measures for fitness function such as Mean Absolute Percent Error (MAPE), Mean Absolute Error (MAE), Mean Squared Error (MSE). However, the proposed relative error measure RE_c_ was more stable in case of extreme values e.g. when the number of infected is close to 0.

**6. Finally, several works pointed out that COVID-19 epidemics, being a human-to-human driven disease, evolves locally [1]. The infected persons spread the disease to close susceptible persons, not to any suspected person in the entire country. Hence, the model will not work at the national level. A good evolution of your regression analysis is working at some regional level. Additionally, working at this level, the outflow infections can be easily interpreted as internal migration.**

**[1] Some papers:**

**Arenas, Alex, et al. "Modeling the spatiotemporal epidemic spreading of COVID-19 and the impact of mobility and social distancing interventions." Physical Review X 10.4 (2020): 041055.**

**Cardoso, Ben-Hur Francisco, and Sebastián Gonçalves. "Universal scaling law for human-to-human transmission diseases." EPL (Europhysics Letters) 133.5 (2021): 58001.**

Abstract models, that were used, consider the possibility of spreading the disease to arbitrary person in the population which is certain limitation of used models. In reality, the infected persons spread the disease to close susceptible persons.

Therefore, one should consider, that human-to-human driven diseases, evolves locally. For better fitting results spatiotemporal spreading of infections should be considered, however, with the available limited dataset, the development of more detailed model could not be performed. This is also the reason that most predictions were based on abstract models such as the ones used in our study.

Development of spatiotemporal models should be addressed in the future with the usage of new technologies, that enable the tracking and monitoring of health data of everyone. This on the other hand raises the question of privacy just to mention one.

The main contribution of the paper lies in the identification of possible new mechanisms that underlies the Covid-19 spread by examining different model structures.

In the discussion section, the following part was added: “One should be aware, that the spread of infectious disease inevitably involves spatially connected dynamics, which could be modelled in more details. Several studies pointed out that COVID-19 epidemics, being a human-to-human driven disease, evolves locally [29, 30]. The infected persons spread the disease to close susceptible persons, not to any suspected person in the entire country. One should note, that this is the limitation of the presented models, which might be used on an abstract, aggregate level.”

Reviewer #2

**1) Concerning the original Bass diffusion model, it is possible to become infected by external sources. Is it reasonable for COVID-19 transmission? Nowadays it is assumed that its transmission is mainly by infected people; the risk of transmission by contaminated surfaces is considered very low. I suggest the authors present some references concerning that discussion, from the epidemiological point of view since it is a central point of the manuscript.**

We added references to the following part of the discussion: “This would suggest that a significant part of the dynamics is not due to the contact between the susceptible and infected [31], but because of some external factors [32].“

The following part has been added in the discussion: “According to [33], “The ability of SARS-CoV-2 to remain viable longer on surfaces taken together with its higher virulence in establishing an infection makes it very likely that this coronavirus uses other modes of transmission in addition to respiratory droplets”. This could be one explanation of the observed higher efficiency of the modified models.“

31 Jayaweera, M., Perera, H.R., Gunawardana, B., & Manatunge, J. (2020). Transmission of COVID-19 virus by droplets and aerosols: A critical review on the unresolved dichotomy. Environmental Research, 188, 109819 – 109819, <https://doi.org/10.1016/j.envres.2020.109819>
32 Lewis, D. (2021). COVID-19 rarely spreads through surfaces. So why are we still deep cleaning? Nature, 590 7844, 26-28, <https://doi.org/10.1038/d41586-021-00251-4>
33 Galbadage, T., Peterson, B.M., & Gunasekera, R.S. (2020). Does COVID-19 Spread Through Droplets Alone? Frontiers in Public Health, 8, <https://doi.org/10.3389/fpubh.2020.00163>

**2) Assuming the hypothesis that infection may occurs without contact with infected people, your results show that the modified models, inspired in Bass diffusion model, fit data better than the original ones. My other main question is related to the parametrization: beside the data and the fitting methods, the parameters obey ranges of values related to the dynamics of the disease. May the authors justify the parameters’ ranges of values used in the simulations for the set of considered countries? For instance, $\beta$ varies from 0 to 4 and $\theta$ varies from 0 to 0.1, but in the discussion the authors conclude that “significant part of the infection is due to external sources. May the authors discuss this point?**

The ranges for $\sigma$ and $\gamma$ were chosen according to preliminary experiments and literature [26], where transmission from exposed to infected and from infected to recovered were determined between 3 and 14 days. For $\beta$ the search range was set from 0 to 4 according to preliminary experiments and literature, for example in [27] the $\beta$ parameter was set to from 1.5 to 3.

To estimate whether the ranges of the parameters search are appropriate, the boxplots were drawn for the $\beta$ parameter for all considered models. The $\beta$ parameter was chosen, as it is present in all models, and has significant influence, as it is responsible for new infections caused by contacts between infected and susceptible. Figure 16 shows box plots for all models, the parameter values were taken from all 10 runs on all countries.

26 Chu, J. A statistical analysis of the novel coronavirus (COVID-19) in Italy and Spain. *PLoS ONE,* 2021, *16*, <https://doi.org/10.1371/journal.pone.0249037>
27 Schmitt, F.G. An algorithm for the direct estimation of the parameters of the SIR epidemic model from the I(t) dynamics. *European Physical Journal plus,* 2022*,* 137, <https://doi.org/10.1140/epjp/s13360-021-02237-7>

**3) In relation to figures 7, 8, 9 and 10, where the usual models are compared to their modified versions, wasn’t it possible to reach the same curve of the modified versions changing the parameter values of the usual models? If it is possible, what are the arguments not to do that procedure? For instance, in Figure 9, changing the value of $\beta$ of SIR model, the curve of eSIR may be reached.**

For particular cases, we could tune the parameters of the standard model to closely fit to the modified model, but it is not possible to get equal response, especially for more complex models.

This could also be examined by analytical solutions which, for the simple cases, might be obtained. The analytical solutions are different thus it is not possible to get exactly the same response, however close fit could be possible.

**4) The authors say that they eliminate countries as Denmark, Brazil, Uganda, Vietnam, Poland and Northern Macedonia from their analysis “due to peculiarities in the data”. It is supposed that those peculiarities are different for the cited countries. However, at least in the case of Brazil, it would be relevant to explain its exclusion since it was the second country in the number of reported cases during the first-wave of COVID-19, and for part of the period, it was the first country in the number of deaths due to the disease.**

Brazil data did not exercise the clear border between first and second wave same as some other listed countries. As long as our goal was to work with the first wave it was not suitable for the analysis. The data of mentioned countries is still valid though not suitable to examine the dynamics of the first wave, which should be clearly present in data.

**5) Looking at the errors of the results for 61 countries, in table 3, for different models, I think that the authors may include, beside the table, a graphical representation of the errors in order to provide a better visualization for comparison between the countries. Due to the number of countries (61) and the number of models (8), the graphics of errors may be presented in a supplementary material.**

We have added Figure 15, where the SEIR model is chosen as baseline, and other models’ relative errors are scaled. Also, the countries are ordered for better visibility.

**6) It was curious that the model with unreported cases do not provide good results. Is it associated to the number of parameters or to its variability. Do the authors have any justification for that result?**

In order to compensate the complexity of optimization problems in cases like SEIUR model, the number of generations of DE was increased to 600. According to Figure 15, the models with unreported cases perform similarly to the SEIR and eSEIR models, although a little larger error values were observed. Our justification is that there are several local optima in the search space, and the optimization process converges to one of them. These local optima generate similar dynamics, each providing close fit with the data. The complexity of models with unreported cases adds to the variability, and significantly increases the number of possible local optima, whereas for models without it the optimization converges faster.

**7) I suggest the authors include the analytical expressions for the basic reproduction number for all models in order to make the comparison easier for the reader.**

We have provided the reproduction number $R0=\beta/\gamma$ only for the SIR model based on the estimated parameters. Determination of R0 number for SEIR and SEIUR models is challenging according to several studies (Heng et al., 2020) and it was not in the main focus of our study. For SEIR and SEIUR models there are analytical solutions, however, they require the death rate to be considered, which was not the case in our study. If the death rate is set to 0, these equations simplify to $R0=\beta/\gamma$, however, as $\beta$ and $\gamma$ are in different equations, the results of such calculation are often unstable, i.e. R0 could be as large as 30, or as small as 0.3.

Heng, K., & Althaus, C.L. (2020). The approximately universal shapes of epidemic curves in the Susceptible-Exposed-Infectious-Recovered (SEIR) model. *Scientific Reports, 10*.

**8) I recommend that the authors include in caption of figure 6 the fitted values of parameters that lead to the curves exhibited in figure 6. It may help the reproduction of the results**

We have added Table 7 with all parameter values to reproduce the graphs in Figure 17 (the figure numbers were changed). Added: “The values in Tables 7 and 8, combined with the description of all models can be used to reproduce the performed experiments.”
